# Aggressive Local Treatment Improves Survival in Stage IV Breast Cancer With Synchronous Metastasis

**DOI:** 10.3389/fonc.2020.522580

**Published:** 2020-11-16

**Authors:** Chen-Lu Lian, Li-Yi Guo, Lei Zhang, Jun Wang, Jian Lei, Li Hua, Zhen-Yu He, San-Gang Wu

**Affiliations:** ^1^ Department of Radiation Oncology, Cancer Hospital, The First Affiliated Hospital of Xiamen University, Xiamen, China; ^2^ The Sixth People’s Hospital of Huizhou, Affiliated Huiyang Hospital of Southern Medical University, Huizhou, China; ^3^ Department of Radiation Oncology, Sun Yat-sen University Cancer Center, State Key Laboratory of Oncology in South China, Collaborative Innovation Center of Cancer Medicine, Guangzhou, China; ^4^ Department of Obstetrics and Gynecology, The First Affiliated Hospital of Xiamen University, Xiamen, China

**Keywords:** breast cancer, distant metastasis, treatment, prognosis, radiotherapy

## Abstract

**Introduction:**

To investigate the effect of local treatment strategy on survival outcome in *de novo* stage IV breast cancer patients who received chemotherapy.

**Methods:**

We identified stage IV breast cancers that presented with synchronous metastasis from the Surveillance, Epidemiology, and End Results database. Binomial logistic regression, Kaplan–Meier survival curves, propensity score matching (PSM), and multivariate Cox regression model were used for statistical analyses.

**Results:**

We identified 5,374 patients in total, including 2,319 (43.2%), 2,137 (39.8%), and 918 (17.1%) patients who received surgery alone, surgery+radiotherapy, and radiotherapy alone, respectively. The probability of patients receiving surgery alone decreased over time, and the probability of patients receiving radiotherapy alone increased over time. However, no significant difference was observed in the probability of patients receiving postoperative radiotherapy (P = 0.291). The 3-year breast cancer-specific survival (BCSS) in patients treated with surgery alone, radiotherapy alone, and surgery+radiotherapy was 57.1, 35.9, and 63.9%, respectively (P < 0.001). The local treatment strategy was the independent prognostic factor related to BCSS. Using surgery alone as the reference, radiotherapy alone was related to lower BCSS (P < 0.001), while additional radiotherapy after surgery improved BCSS (P < 0.001). Similar results were observed using PSM.

**Conclusions:**

Compared to radiotherapy alone, surgery to the primary site may confer a survival benefit in stage IV breast cancer with synchronous metastasis, and additional postoperative radiotherapy further improves outcome after primary tumor removal. Local treatment can only be an option in highly selected patients with *de novo* stage IV disease in the treatment guidelines. More prospective studies are needed to investigate the role of local management for this patient subset.

## Background

Breast cancer remains the leading cause of malignancy in women worldwide, with approximately two million new cases diagnosed in 2018 (1). About 3–5% of newly diagnosed breast cancer cases are stage IV disease with synchronous metastasis (*de novo* stage IV disease) (2–4). Although related to poor outcomes, advances in systemic therapies against breast cancer such as taxane-based chemotherapy, targeted therapies, and endocrine therapy have improved the survival outcomes of stage IV patients (5). Two recent studies have indicated that the prognosis has improved over the past three decades in this patient subset (6, 7).

Traditional management in this patient subset comprises systemic therapy, with additional surgery or radiotherapy to control locoregional symptoms. However, four recent randomized trials that investigated prognosis after surgery in *de novo* stage IV breast cancer reported conflicting results (8–11). Several retrospective studies have shown a survival advantage with locoregional treatment, including surgery or radiotherapy to the primary site (12–21). The rationale for proceeding with additional surgery or radiotherapy includes the possibility of increasing the effectiveness of chemotherapy, reducing the total tumor burden, restoring immunity, eliminating breast cancer stem cells, and decreasing the likelihood of resistant disease, which may lower the metastatic potential of the primary tumor (22–24). These observations suggest that locoregional intervention to primary tumors may improve outcome in stage IV breast cancer with synchronous metastasis.

In current clinical practice, approximately half of the patients with *de novo* stage IV disease were treated with local surgery, because it was associated with better local control and longer survival times in retrospective studies (19, 25, 26). The consensus from the Third International Consensus Conference for Advanced Breast Cancer suggests that surgery to the primary site can be considered in selected patients, particularly to improve the quality of life (27). However, the survival benefit of radiotherapy in these patients has been rarely investigated (13, 20, 21). In addition, it is worth speculating whether postoperative radiotherapy could improve survival, as this has shown conflicting results in the past (13, 18, 19, 21). Therefore, we explored the existing real-world data from the Surveillance, Epidemiology, and End Results (SEER) program to assess the outcomes of different local treatment strategies including surgery alone, radiotherapy alone, and surgery+radiotherapy for patients with stage IV breast cancer with synchronous metastasis.

## Methods and Materials

### Patients

Patient data were selected from the SEER database that includes patient information regarding clinical cancer incidence, demographics, clinicopathological characteristics, the first course of treatment including surgery, radiotherapy, and chemotherapy, and vital status from 18 registries, which represents approximately 28% of the population of the United States (28). We identified *de novo* stage IV breast cancer patients treated with surgery alone, radiotherapy alone, or surgery and radiotherapy in addition to chemotherapy, between 2004 and 2012. The following patients were excluded: those with no pathologic diagnosis, those with non-invasive ductal carcinoma or invasive lobular carcinoma, those that did not undergo external beam radiation, and those with unavailable data regarding ethnicity, grade, tumor size, nodal status, estrogen receptor (ER), and progesterone receptor (PR) status. The Institutional Review Board waived the need for informed consent because anonymized patient data from the SEER database was used.

### Measures

We identified the following variables of interest: age, ethnicity, grade, histology, T stage, N stage, ER status, PR status, and local treatment procedures. T and N category was determined based on the seven edition of the UICC/AJCC staging system. The primary outcome of this study was breast cancer-specific survival (BCSS), which was calculated as the time from the initial diagnosis to the date of breast cancer-specific death or last follow-up.

### Statistical Analysis

The distribution differences among locoregional treatment procedures and patient information were compared using the chi-square test. Predictors of receipt of locoregional treatment procedures were analyzed using binomial logistic regression. A 1:1 propensity score matching (PSM) method was performed by logistic regression to balance the above patient demographic and clinicopathological characteristics to reduce the potential baseline selection bias. The BCSS rate was assessed using the Kaplan–Meier method, and the effect of locoregional treatment procedures on BCSS was analyzed using the log-rank test. The independent prognostic indicators associated with BCSS were determined using multivariate Cox regression models with Backward Wald. IBM SPSS version 22.0 (IBM Corp., Armonk, NY, USA) was used for all statistical analyses, and a P < 0.05 was considered to indicate statistical significance.

## Results

### Patient Characteristics

**Figure 1:**
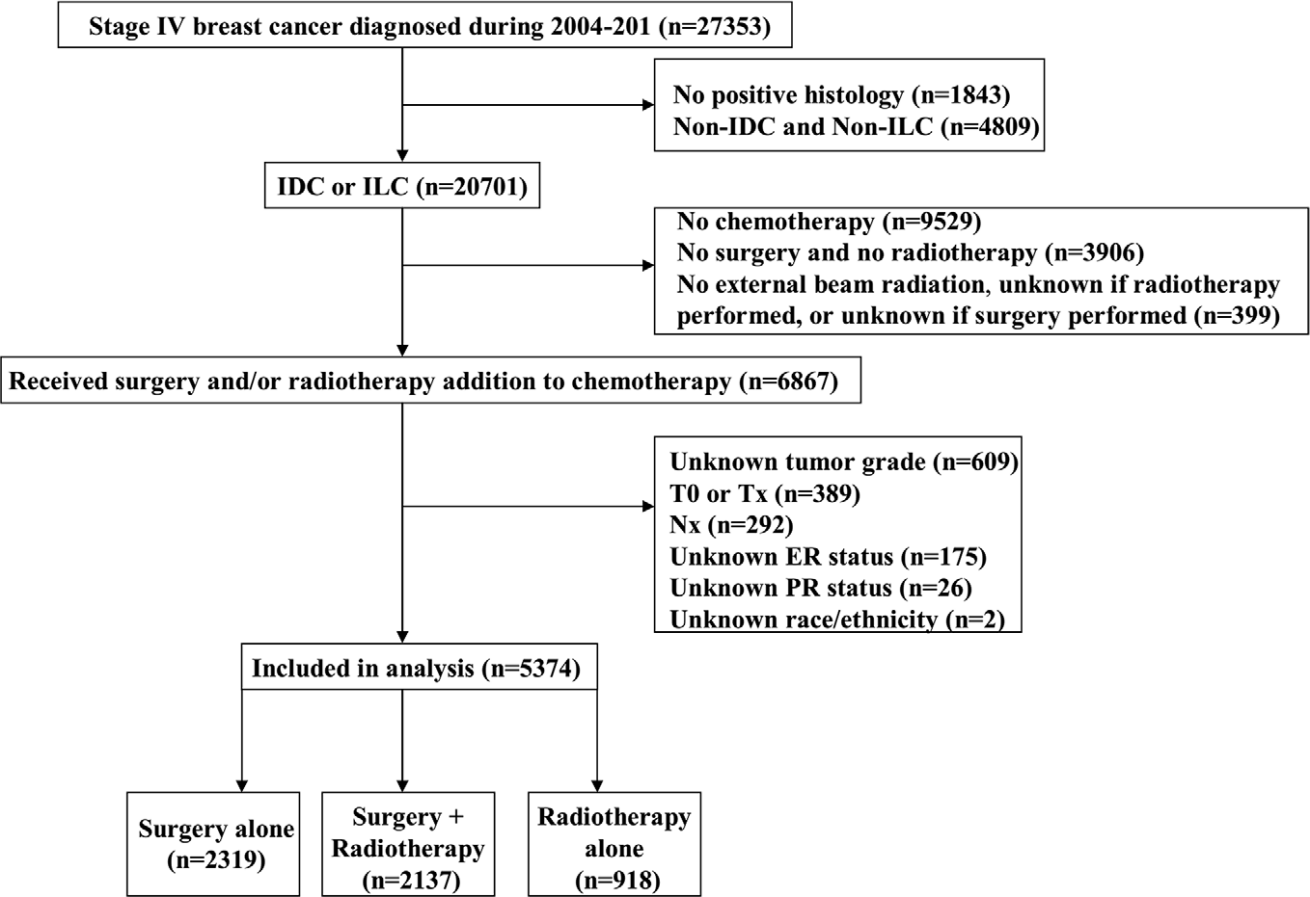
The patient selection flowchart of the study.

We identified 5,374 patients from the SEER database in this study. A flow-chart of patient selection is shown in **Figure 1**. Of these patients, 93.2% (n = 5,006) had invasive ductal carcinoma, 81.6% (n = 4,387) had node-positive disease, 76.3% (n = 4,102) were aged <65 years, 64.3% (n = 3,454) had ER-positive disease, 62.1% (n = 3,339) were non-Hispanic White, and 60.8% (n = 3,272) had poorly differentiated/undifferentiated disease. In addition, approximately 50% of patients had stage T3-4 disease. A total of 4,456 patients underwent surgical treatment, and 48.0% (n = 2,137) of them were treated with postoperative radiotherapy, while 918 patients received radiotherapy alone. The patient baseline characteristics are listed in **Table 1**.

**Table 1:** Patients’ baseline characteristics.

Variables	n	S alone (%)	RT alone (%)	S + RT (%)	P
Age (years)					
<65	4,102	1,707 (73.6)	691 (75.3)	1,704 (79.7)	<0.001
≥65	1,272	612 (26.4)	227 (24.7)	433 (20.3)	
Ethnicity					
Non-Hispanic White	3,339	1,465 (63.2)	521 (56.8)	1,353 (63.3)	0.008
Non-Hispanic Black	946	405 (17.5)	193 (21.0)	348 (16.3)	
Hispanic (all ethnicities)	641	272 (11.7)	115 (12.5)	254 (11.9)	
Other	448	177 (7.6)	89 (9.7)	182 (8.5)	
Grade					
Well-differentiated	285	118 (5.1)	57 (6.2)	110 (5.1)	<0.001
Moderately differentiated	1,817	711 (30.7)	374 (40.7)	732 (34.3)	
Poorly/undifferentiated	3,272	1,490 (64.3)	487 (53.1)	1,295 (60.6)	
Histology					
IDC	5,006	2,148 (92.6)	866 (94.3)	1,992 (93.2)	0.219
ILC	368	171 (7.4)	52 (5.7)	145 (6.8)	
Tumor stage					
T1	774	361 (15.6)	115 (12.5)	298 (13.9)	<0.001
T2	1,916	913 (39.4)	211 (23.0)	792 (37.1)	
T3	991	448 (19.3)	154 (16.8)	389 (18.2)	
T4	1,693	597 (25.7)	438 (47.7)	658 (30.8)	
Nodal status					
N0	987	451 (19.4)	223 (24.3)	313 (14.6)	<0.001
N1	2,105	865 (37.3)	450 (49.0)	790 (37.0)	
N2	1,029	468 (20.2)	94 (10.2)	467 (21.9)	
N3	1,253	535 (23.1)	151 (16.4)	567 (26.5)	
ER status					
Negative	1,920	918 (39.6)	322 (35.1)	680 (31.8)	<0.001
Positive	3,454	1,401 (60.4)	596 (64.9)	1,457 (68.2)	
PR status					
Negative	2,702	1,235 (53.3)	464 (50.5)	1,003 (46.9)	<0.001
Positive	2,672	1,084 (46.7)	454 (49.5)	1,134 (53.1)	

IDC, invasive ductal carcinoma; ILC, invasive lobular carcinoma; ER, estrogen receptor; N, nodal; PR, progesterone receptor; RT, radiotherapy; S, surgery; T, tumor.

### Trends of Local Treatment Receipt

**Figure 2:**
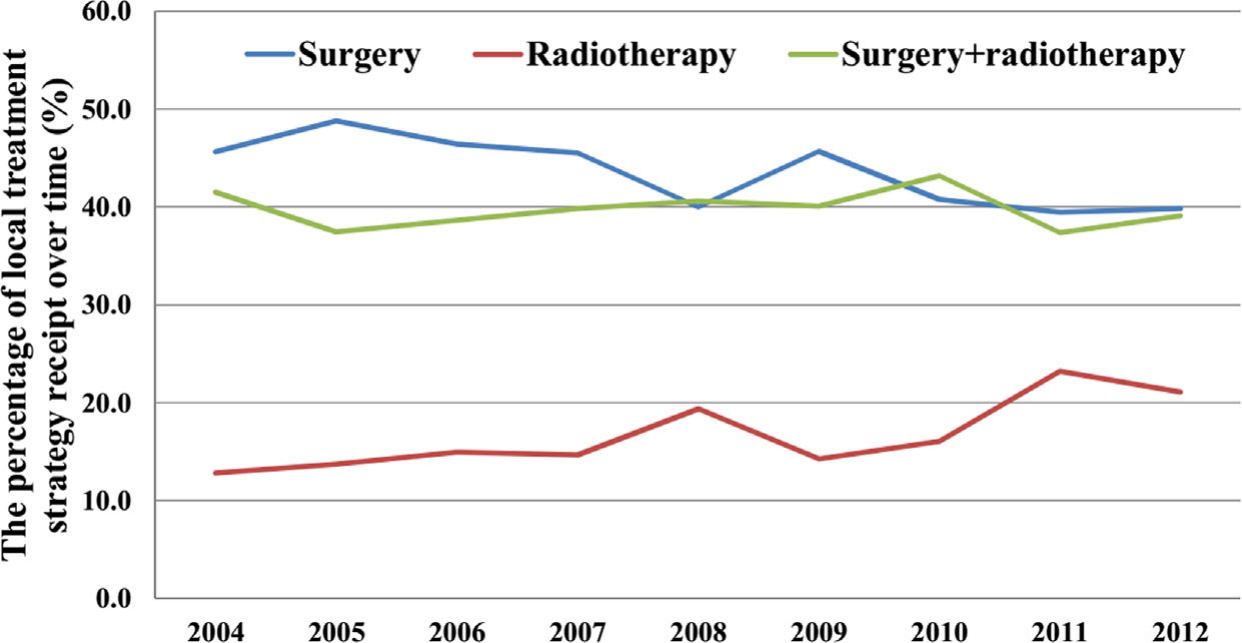
The probability of receiving different local treatment strategies over time.

The use of surgery alone decreased from 45.7% in 2004 to 39.8% in 2012 (P < 0.001). However, no significant difference was observed in the probability of patients receiving postoperative radiotherapy (41.6% in 2004 *vs.* 39.5% in 2012, P = 0.291). Moreover, the probability of receiving radiotherapy alone showed an increase over time, from 12.8% in 2004 to 21.1% in 2012 (P < 0.001). **Figure 2** shows the probability of receiving different local treatments over time.

### Predictors for Receipt of Local Treatment

Using binomial logistic regression (**Table 2**), we found that ethnicity, grade, T stage, and N stage were independent predictors of radiotherapy receipt. Patients with non-Hispanic Black and other ethnicities, lower tumor grade, larger tumor size, and node-negative disease were more likely to be treated with radiotherapy alone. In addition, age, T stage, N stage, and ER status were independent predictors of postoperative radiotherapy receipt. Patients with younger age, T4 stage, and node-positive and ER-positive disease were more likely to receive postoperative radiotherapy.

**Table 2:** Predictive factors for receipt of local treatment.

Variables	RT alone *vs.* S ± RT			S + RT *vs.* S alone		
	OR	95% CI	P	OR	95% CI	P
Age (years)						
<65	1			1		
≥65	1.057	0.889–1.257	0.532	0.698	0.606–0.804	<0.001
Ethnicity						
Non-Hispanic White	1			1		
Non-Hispanic Black	1.367	1.129–1.654	0.001	0.905	0.767–1.068	0.237
Hispanic (All ethnicities)	1.188	0.944–1.495	0.143	0.98	0.811–1.184	0.834
Other	1.332	1.027–1.726	0.031	1.055	0.845–1.318	0.636
Grade						
Well-differentiated	1			1		
Moderately differentiated	0.982	0.712–1.356	0.914	1.032	0.775–1.374	0.830
Poorly/undifferentiated	0.635	0.462–0.873	0.005	0.907	0.680–1.210	0.506
Histology						
IDC	1			1		
ILC	0.796	0.578–1.097	0.163	0.847	0.664–1.081	0.182
Tumor stage						
T1	1			1		
T2	0.852	0.663–1.095	0.212	0.995	0.828–1.196	0.960
T3	1.367	1.039–1.797	0.025	0.982	0.795–1.212	0.863
T4	2.726	2.140–3.472	<0.001	1.296	1.066–1.577	0.009
Nodal status						
N0	1			1		
N1	0.776	0.640–0.941	0.010	1.24	1.038–1.481	0.018
N2	0.282	0.216–0.370	<0.001	1.366	1.121–1.665	0.002
N3	0.372	0.292–0.472	<0.001	1.482	1.223–1.796	<0.001
ER status						
Negative	1			1		
Positive	1.013	0.820–1.252	0.903	1.427	1.259–1.617	<0.001
PR status						
Negative	1			1		
Positive	0.941	0.770–1.150	0.551	1.040	0.883–1.226	0.637

CI, confidence interval; IDC, invasive ductal carcinoma; ILC, invasive lobular carcinoma; ER, estrogen receptor; N, nodal; OR, odds ratio; PR, progesterone receptor; RT, radiotherapy; S, surgery; T, tumor.

### Survival and Prognostic Analyses

**Figure 3:**
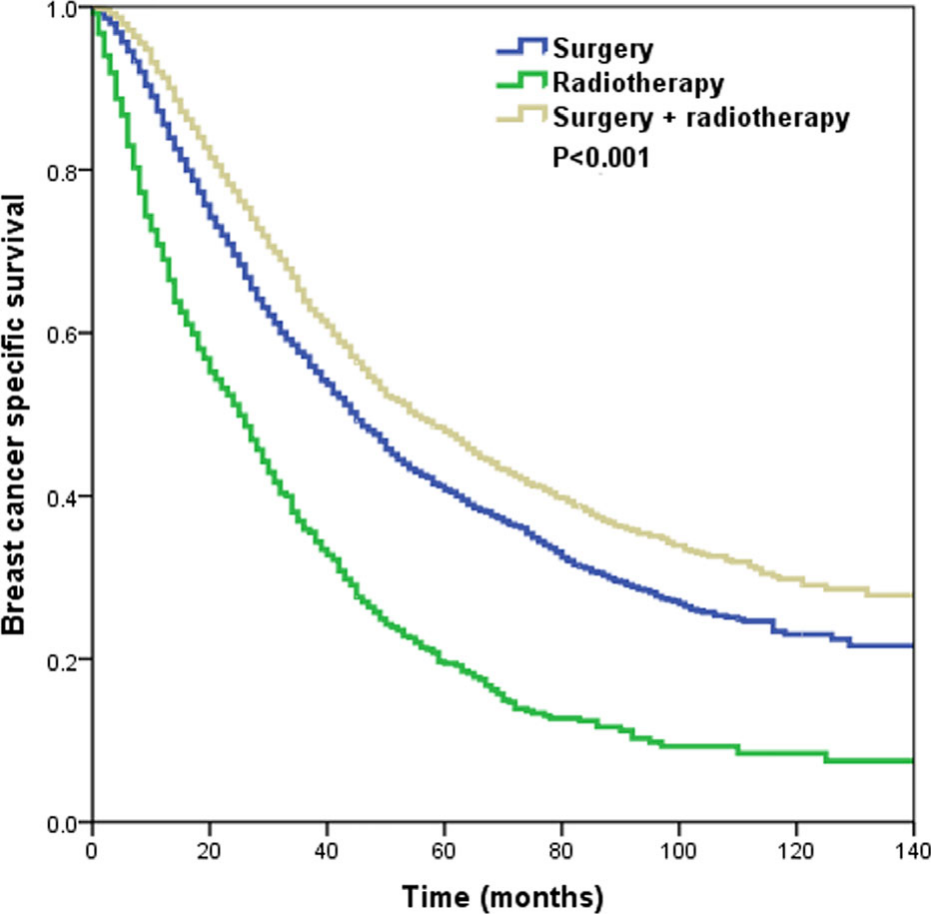
The Kaplan–Meier curves of breast cancer-specific survival by different local treatment strategies before propensity score matching.

With a median follow-up of 37 months (range, 0–143 months), a total of 3,727 patients died, including 3,317 patients who died with breast cancer. The 3- and 5-year BCSS was 56.3 and 40.2%, respectively. The 3-year BCSS in patients that underwent surgery alone, radiotherapy alone, and surgery+radiotherapy was 57.1, 35.9, and 63.9%, respectively, with a median survival time of 45, 25, and 55 months, respectively (P < 0.001) (**Figure 3**).

In the multivariate Cox regression analysis (**Table 3**), local treatment strategy also served as an independent prognostic factor related to BCSS. Using surgery alone as the reference, radiotherapy alone was related to lower BCSS (hazard ratio [HR]: 1.966, 95% confidence interval [CI]: 1.788–2.162, P < 0.001), while additional radiotherapy after surgery improved BCSS (HR: 0.829, 95% CI: 0.767–0.896, P < 0.001). In addition, age, ethnicity, grade, histology, T stage, N stage, and hormone receptor status were the prognostic factors related to BCSS.

**Table 3:** Multivariate analysis on prognostic indicators associated with breast cancer-specific survival before propensity score matching.

Variables	HR	95% CI	P
Age (years)			
<65	1		
≥65	1.171	1.081–1.269	<0.001
Ethnicity			
Non-Hispanic White	1		
Non-Hispanic Black	1.321	1.208–1.444	<0.001
Hispanic (all ethnicities)	1.023	0.917–1.140	0.686
Other	0.816	0.714–0.933	0.003
Grade			
Well-differentiated	1		
Moderately differentiated	1.120	0.937–1.338	0.214
Poorly/undifferentiated	1.418	1.188–1.693	<0.001
Histology			
IDC	1		
ILC	1.187	1.033–1.363	0.015
Tumor stage			
T1	1		
T2	1.180	1.051–1.326	0.005
T3	1.360	1.196–1.546	<0.001
T4	1.565	1.390–1.761	<0.001
Nodal status			
N0	1		
N1	0.881	0.798–0.974	0.013
N2	0.955	0.820–1.076	0.436
N3	1.064	0.954–1.187	0.267
ER status			
Negative	1		
Positive	0.752	0.683–0.827	<0.001
PR status			
Negative	1		
Positive	0.712	0.648–0.782	<0.001
Treatment			
Surgery alone	1		
Radiotherapy alone	1.966	1.788–2.162	<0.001
Surgery + radiotherapy	0.829	0.767–0.896	<0.001

CI, confidence interval; IDC, invasive ductal carcinoma; ILC, invasive lobular carcinoma; ER, estrogen receptor; HR, hazard ratio; N, nodal; PR, progesterone receptor; RT, radiotherapy; S, surgery; T, tumor.

**Figure 4:**
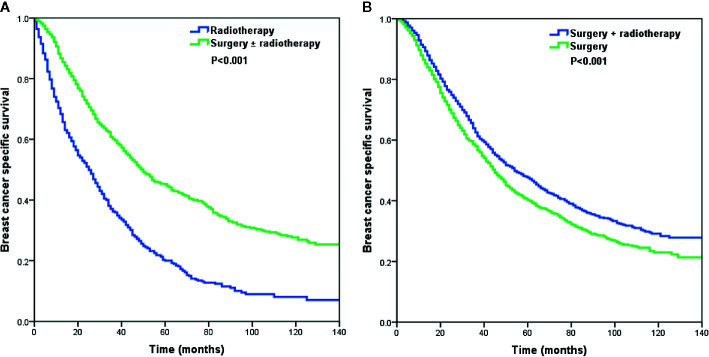
The Kaplan–Meier curves of breast cancer-specific survival by different local treatment strategies after propensity score matching **(A)** surgery ± radiotherapy *vs.* radiotherapy alone; **(B)** surgery+radiotherapy *vs.* surgery alone).

Using PSM, a total of 792 pairs were completely matched between the surgery ± radiotherapy and radiotherapy alone cohorts. In addition, 1,469 pairs were completely matched between surgery alone and surgery+radiotherapy cohorts. After adjustment of age, ethnicity, grade, histology, T stage, N stage, and hormone receptor status, the results confirmed that patients who received radiotherapy alone had lower BCSS than those who were treated with surgery ± radiotherapy (HR: 2.135, 95% CI: 1.889–2.412, P < 0.001) (Model 1) (**Table 4**). Moreover, patients who received postoperative radiotherapy had better BCSS than those treated with surgery alone (HR: 0.814, 95% CI: 0.742–0.893, P < 0.001) (Model 2) (**Table 4**). The survival curves in the two cohorts are shown in **Figure 4**.

**Table 4:** Multivariate analysis on prognostic indicators associated with breast cancer-specific survival after propensity score matching.

Variables	Model 1			Model 2		
	HR	95% CI	P	HR	95% CI	P
Age (years)						
<65	1			1		
≥65	1.160	1.002–1.343	0.047	1.035	0.919–1.167	0.570
Ethnicity						
Non-Hispanic White	1			1		
Non-Hispanic Black	1.302	1.118–1.517	0.001	1.511	1.334–1.711	<0.001
Hispanic (all ethnicities)	1.132	0.930–1.377	0.217	1.059	0.904–1.240	0.477
Other	0.727	0.565–0.936	0.013	0.890	0.727–1.091	0.264
Grade						
Well-differentiated	1			1		
Moderately differentiated	1.219	0.866–1.714	0.256	1.597	1.110–2.296	0.012
Poorly/undifferentiated	1.393	0.987–1.965	0.059	2.083	1.445–3.004	<0.001
Histology						
IDC	1			1		
ILC	0.913	0.608–1.371	0.660	1.647	1.291–2.101	<0.001
Tumor stage						
T1	1			1		
T2	1.158	0.931–1441	0.187	1.011	0.863–1.185	0.892
T3	1.139	0.880–1.423	0.361	1.309	1.095–1.564	0.003
T4	1.373	1.117–1.688	0.003	1.545	1.311–1.820	<0.001
Nodal status						
N0	1			1		
N1	0.859	0.733–1.007	0.061	0.905	0.783–1.045	0.173
N2	0.984	0.776–1.246	0.892	0.979	0.833–1.151	0.797
N3	1.054	0.860–1.293	0.611	1.138	0.975–1.328	0.102
ER status						
Negative	1			1		
Positive	0.663	0.558–0.789	<0.001	0.822	0.717–0.944	0.005
PR status						
Negative	1			1		
Positive	0.734	0.622–0.866	<0.001	0.721	0.629–0.827	<0.001
Treatment						
Surgery ± radiotherapy	1			—		
Radiotherapy	2.135	1.889–2.412	<0.001	—	—	—
Treatment						
Surgery	—			1		
Surgery + radiotherapy	—	—	—	0.814	0.742–0.893	<0.001

CI, confidence interval; IDC, invasive ductal carcinoma; ILC, invasive lobular carcinoma; ER, estrogen receptor; HR, hazard ratio; N, nodal; PR, progesterone receptor; RT, radiotherapy; S, surgery; T, tumor.

## Discussion

In the current study, we used the SEER database to investigate whether aggressive local treatment improves survival in stage IV breast cancer with synchronous metastasis. Our results showed that local surgery was related to better BCSS than radiotherapy alone, and additional postoperative radiotherapy further improved BCSS than surgery alone.

*De novo* stage IV breast cancer is a relatively rare disease, and most patients were treated with systemic therapy only. The efficacy of local treatment, such as surgery and/or radiotherapy remains controversial. Thus, there were significant differences in the distribution regarding local treatment strategies in these patients. In a study by Choi et al. that included 245 patients, 82 patients received locoregional treatment and systemic therapy, and 32.9, 11.0, and 56.1% of them received surgery alone, radiotherapy alone, and surgery+radiotherapy, respectively (18). Another study from the British Columbia Cancer Agency (n = 378) indicated that surgery was the most common treatment procedure (78.3%), with only 13.9% (n = 41) patients receiving radiotherapy in the surgery cohort and 21.7% patients receiving radiotherapy alone (19). However, another study from Le Scodan et al. that included 320 patients treated with locoregional treatment showed that 78% (n = 249) received radiotherapy alone, 71 (22.2%) received surgery, and 57.7% had additional radiotherapy (13). In our study, the distribution of the types of local treatment was 43.2, 17.1, and 38.8 in the surgery alone, radiotherapy alone, and surgery+radiotherapy, respectively. There was no consensus regarding the locoregional treatment in this patient subset. Therefore, the different distribution of locoregional treatment might reflect the different clinical practices in various institutions.

To our best knowledge, no study has so far assessed changes to local treatment patterns in *de novo* stage IV breast cancer over time. In this study, we additionally investigated the relationship between the patterns of local treatment and the time of diagnosis. Our results showed that from 2004 to 2012, patients who received surgery alone decreased by 5.9% (45.7 *vs.* 39.8%), while those that received radiotherapy alone increased by 8.3% (12.8 *vs.* 21.1%). The main reason for the changing trends of local treatment remains unclear. A possible explanation is that systemic treatments for breast cancer patients, including chemotherapy, targeted therapy, and endocrine therapy, have made significant progress, and the outcomes have improved (5), which may have reduced the use of aggressive treatments, including surgery.

Although an improvement in median survival was observed with upfront local surgery for *de novo* stage IV breast cancer in the MF07-01 trial (9), there were three randomized trials, including TATA memorial study, E2108 trial, and ABCSG-28 POSYTIVE trial, which investigated local therapy for *de novo* stage IV breast cancer and indicated that additional local therapy to optimal systemic therapy did not improve survival outcomes than those treated with optimal systemic therapy alone (8, 10, 11). In the current clinical practice, approximately half of patients with *de novo* stage IV breast cancer were treated with local therapy (15, 16, 19, 21, 25, 26). Therefore, according to our findings, if the clinicians decide to use local treatment in select cases, it appears that surgery+radiotherapy is better than those with radiotherapy or surgery alone.

Results regarding the predictive factors of receipt of radiotherapy alone were contradictory. A study by Le Scodan et al. included patients who received radiotherapy alone or no local treatment, patients with small tumor size, lower nodal stage, non-visceral metastases, and received a combination of endocrine treatment and chemotherapy were more likely to received radiotherapy (13). Another study from the Institut Gustave Roussy Breast Cancer Database showed that patients with large tumor size, higher tumor grade, advanced nodal stage, and higher tumor burden were more likely to be included in the radiotherapy alone than surgery ± radiotherapy cohort (21). Our results also showed that patients with favorable prognostic factors, including lower tumor grade and node-negative disease were more likely to received radiotherapy alone. However, patients with larger tumor size also had a higher chance of receiving radiotherapy alone compared to surgery cohort. The results from a meta-analysis showed that patients with larger tumor size were less likely to undergo surgery (20). Thus, locoregional radiotherapy might be a reasonable choice for patients with larger tumor size if locoregional management was to be performed. However, our study showed that radiotherapy alone had the worst survival.

Although the efficacy of local treatment in these patients showed contradictory results in prospective studies (8–11), current retrospective studies with large cohorts had suggested that local treatment could improve the survival of this patient subset (12–21). However, most studies are mainly based on surgical treatment, and there are currently no prospective studies to compare the role of radiotherapy and surgery. A study by Le Scodan et al. showed that patients in the radiotherapy cohort had better 3-year overall survival (OS) (43.4 *vs.* 26.7%, P < 0.001) than patients who did not undergo any local treatment (13). They suggested that locoregional radiotherapy may be an effective alternative to surgery. However, more patients who are treated with radiotherapy alone had smaller tumor size, lower nodal burden, bone-only metastases, and less visceral organ involvement, and more received endocrine therapy (13). Two recent studies from the Institut Gustave Roussy Breast Cancer Database and the British Columbia Cancer Agency showed comparable survival outcomes between surgery ± radiotherapy and radiotherapy-alone cohorts when adjusted for prognostic factors (19, 21). However, patients who received surgery ± radiotherapy were less likely to be treated with systemic therapies (55 *vs.* 99% in surgery ± radiotherapy *vs.* radiotherapy alone, respectively) (21), which may limit the representative value of the study. The study by Choi et al. included 245 patients, wherein 90% were treated with chemotherapy, and patients with surgery ± radiotherapy had significantly higher locoregional-free survival (LRFS) and OS rates than the radiotherapy-only cohort (5-year LRFS: surgery+radiotherapy [70%], surgery only [53%], and radiotherapy only [27%]; 5-year OS: surgery+radiotherapy [77%], surgery only [70%], and radiotherapy only [44%]). Moreover, 63.0% of patients received postoperative radiotherapy in the surgery cohort (18). In our large cohort study, all patients were treated with chemotherapy, and patients in the surgery ± radiotherapy cohorts had significantly higher BCSS than those treated with radiotherapy alone before and after PSM, which was similar to Choi et al.’s results (18). Our study indicated that surgery is an acceptable alternative to radiotherapy alone in appropriately selected patients.

There were still large differences in the recommendation for postoperative radiotherapy, ranging from 18.9 to 63.0% (12, 18, 19, 25, 29). Several previous studies have shown comparable LRFS or OS between patients treated with surgery alone and surgery+radiotherapy (18, 19, 25, 29). Our study further indicated that postoperative radiotherapy could improve BCSS in the surgical cohort. The potential interpretation of our results may be with respect to higher tumor burden, including larger tumor size, advanced nodal stage, and higher tumor grade that may have a significant correlation with subsequent locoregional recurrence and distant metastasis. Therefore, postoperative radiotherapy may be an important option, together with local surgical treatment for these patients. Studies from non-metastatic breast cancer have also shown that postoperative radiotherapy can improve locoregional control, distant recurrence, and OS in patients with node-positive lymph nodes (30–32).

Our study has some limitations. First, as with any retrospective study, there exists a possible selection bias with limits any conclusions of direct causative relationships. In addition, we were unable to include targeted therapy and endocrine therapy, given that it was not recorded in the SEER database. Third, the sequence of chemotherapy and local treatment, the timing of local treatment, the evaluation of tumor response to chemotherapy, the recurrence, and distant patterns after local treatment are not recorded in the SEER program. Finally, the dose and target volume of locoregional radiotherapy was also not recorded in the SEER database. The primary strength of this study was that we used a large database series to determine the optimal additional local treatment strategy in *de novo* stage IV breast cancer treated with chemotherapy.

## Conclusion

In conclusion, our study suggests that surgery to primary sites may offer better survival benefit than radiotherapy alone in patients with *de novo* stage IV breast cancer. Additionally, additional postoperative radiotherapy further improves outcomes after primary tumor removal. However, due to lack of important information regarding tumor biology, systemic treatments, and site of metastasis. This study does not provide reliable data on the real impact of local treatments for this patient subset. According to the guidelines from the European School of Oncology and European Society for Medical Oncology (33), local treatment can only be an option in highly selected patients. Therefore, more prospective studies are needed to investigate the role of local management in patients with *de novo* stage IV breast cancer.

## Data Availability Statement

Publicly available datasets were analyzed in this study. This data can be found here: www.seer.cancer.gov.

## Ethics Statement

Ethical review and approval was not required for the study on human participants in accordance with the local legislation and institutional requirements. Written informed consent for participation was not required for this study in accordance with the national legislation and the institutional requirements.

## Author Contributions

C-LL, L-YG, LZ, Z-YH, and S-GW are lead authors who participated in data collection, manuscript drafting, table/figure creation, and manuscript revision. S-GW and Z-YH aided in data collection. JL, LH, and JW are senior authors who aided in drafting the manuscript and manuscript revision. Z-YH and S-GW are the corresponding authors who initially developed the concept and drafted and revised the manuscript. All authors contributed to the article and approved the submitted version.

## Funding

This work was partly supported by the National Natural Science Foundation of China (No. 81872459), the Commission Young and Middle-aged Talents Training Project of Fujian Health Commission (No. 2019-ZQNB-25), the Science and Technology Planning Projects of Xiamen Science & Technology Bureau (No. 3502Z20174070), and the Natural Science Foundation of Guangdong Province (No. 2018A030313666, 2017A030310422).

## Conflict of Interest

The authors declare that the research was conducted in the absence of any commercial or financial relationships that could be construed as a potential conflict of interest.
